# Integrated Analysis of a Ferroptosis-Related LncRNA Signature for Evaluating the Prognosis of Patients with Colorectal Cancer

**DOI:** 10.3390/genes13061094

**Published:** 2022-06-19

**Authors:** Shaohua Xu, Yanjie Zhou, Junyun Luo, Su Chen, Jiahui Xie, Hui Liu, Yirong Wang, Zhaoyong Li

**Affiliations:** 1 Research Institute of Hunan University in Chongqing, Chongqing 401120, China; xushaohua2012@163.com (S.X.); 18373210670@163.com (Y.Z.); 2 Hunan Provincial Key Laboratory of Medical Virology, Institute of Pathogen Biology and Immunology of College of Biology, Hunan University, Changsha 410082, China; junyluo@126.com (J.L.); cschensucs@163.com (S.C.); xiejh@hnu.edu.cn (J.X.); 15704925120@163.com (H.L.); 3 Bioinformatics Center, College of Biology, Hunan University, Changsha 410082, China

**Keywords:** colorectal cancer, ferroptosis, long non-coding RNA, prognostic signature, metastasis

## Abstract

LncRNAs have been well known for their multiple functions in the tumorigenesis, development, and relapse of colorectal cancer (CRC). Accumulating studies demonstrated that the expression of lncRNAs can be regulated by ferroptosis, a biological process that has been revealed to suppress CRC progression. However, the functions and clinical implications of ferroptosis-associated lncRNAs in CRC remain largely unknown. We, herein, aim to construct a prognostic signature with ferroptosis-related lncRNAs for the prognostic estimation of CRC patients. Firstly, we identified the lncRNAs related to ferroptosis based on the RNA-Seq data of CRC from the TCGA database. The univariate and multivariate Cox analyses were then performed to establish a prognostic signature composed of eight ferroptosis-related lncRNAs (AL161729.4, AC010973.2, CCDC144NL-AS1, AC009549.1, LINC01857, AP003555.1, AC099850.3, and AC008494.3). Furthermore, we divided the CRC patients into high- and low-risk groups based on the signature and found the overall survival (OS) of patients in the high-risk group was significantly shorter than that in the low-risk group (*p* = 3.31 × 10^−11^). Moreover, the patients in the high-risk groups had shorter recurrence-free survival (RFS) (*p* = 6.5 × 10^−3^) and disease-free survival (DFS) (*p* = 4.27 × 10^−4^), as well as higher tumor recurrence rate. Additionally, we found that the oncogenic pathways were enriched in the high-risk group, whereas the ferroptosis pathway that probably repressed CRC development was enriched in the low-risk group. In summary, our signature may provide a theoretical foundation for not only accurate judgment for prognosis but also evaluation for recurrence and metastasis in CRC patients.

## 1. Introduction

As one of the most common cancers, colorectal cancer (CRC) is the third most commonly diagnosed cancer and the second leading cause of cancer death worldwide [1]. Statistically, in 2020, the number of new CRC cases exceeded 1.9 million and the number of CRC deaths exceeded 0.9 million, whereas the sum of them accounts for about one-tenth of all cancers [1]. The 5-year survival rate for CRC patients is about 64%, while it is about 12% for metastasized CRC patients [2]. It makes CRC horrific that more than half of CRC patients develop liver metastases [3,4]. Moreover, intrahepatic recurrence usually happens within 3 years after surgical resection of these CRC patients with liver metastases [3] and the 5-year survival rate of the patients is only 20–50% [4]. As the primary prognostic factor, the prognostic performance of the current tumor-node-metastasis (TNM) staging system for CRC patients is still insufficiently accurate [5]. Furthermore, due to the high heterogeneity of CRC, few biological markers can accurately predict its recurrence [6]. Therefore, the identification and validation of new prognostic markers are greatly needed for the precise prognosis of CRC patients.

Ferroptosis, which was first discovered in 2012 [7], is a novel regulatory pattern of cell death that is mediated by iron and lipid peroxidation [8]. Due to its involvement in a variety of biological processes in cancer [9,10,11], ferroptosis has been reported to play a dual role in cancer occurrence and development [8]. On the one hand, ferroptosis can exert its suppressive effect on cancer development [12,13,14] or recurrence [15,16]. On the other hand, ferroptosis may also promote cancer progression in certain circumstances, such as oxidative stress [17,18]. Therefore, ferroptosis induction or inhibition can be employed as a novel therapeutic strategy for certain cancers. Recently, a series of studies have implicated the inhibitory function of ferroptosis in the carcinogenesis and development of CRC [19,20,21,22]. It has been revealed that cellular iron transportation is impaired during colon cancer progression [20]. The ferroptosis induced by resibufogenin treatment is mediated by glutathione peroxidase 4 (GPX4) inactivation and suppresses CRC cell growth and tumorigenesis [19]. Besides, ferroptosis can be triggered by combinative treatment of β-elemene and cetuximab, contributing to the growth retardation and migration suppression of CRC cells with *KRAS* mutations [21]. Collectively, ferroptosis may play a negative regulatory role in the progression of CRC.

Long non-coding RNAs (LncRNAs), a kind of non-protein coding transcript with a length of more than 200 nucleotides [23], are widely involved in a variety of biological processes associated with cancer, including rapid proliferation, apoptosis resistance, angiogenesis, metabolic reprogramming, invasion, metastasis, and so on [23,24,25]. For example, by interacting with heterogeneous nuclear ribonucleoprotein A2B1 (hnRNPA2B1) to upregulate the expression of transcription factor 7 like 2 (TCF7L2) that activates Wnt signaling, lncRNA MIR100HG acts as an inducer of epithelial–mesenchymal transition (EMT), which facilitates cetuximab resistance and metastasis in CRC [26]. Additionally, the aberrant expression of lncRNAs in cancer confers on them the ability to predict the prognosis of patients [23,27,28]. Recently, several studies demonstrated that the expression levels of lncRNAs can be regulated by ferroptosis [29,30,31]. For instance, lncRNA GABPB1-AS1′s expression can be elevated in the hepatocellular carcinoma cells by erastin, which serves as an inducer of ferroptosis [29]. Besides, H19 and NEAT1, two additional lncRNAs, were also up-regulated in cells undergoing ferroptosis [30,31]. Nonetheless, study on lncRNA in connection with ferroptosis in CRC is scarce so far.

Although the ferroptosis-related lncRNA prognostic signatures have been developed for the prognosis evaluation for CRC [32] and clinical outcomes and therapeutic responses for colon cancer patients [33,34], we herein established a prognostic signature based on eight ferroptosis-related lncRNAs that can not only predict OS but also estimate the risk of recurrence and metastasis in CRC patients. Our prognostic signature can categorize CRC patients into high- and low-risk groups and judge a poor prognosis for patients in the high-risk group. Moreover, the signature exhibited broad applicability in various clinical subgroups, independence in evaluating the prognosis of CRC patients, as well as prognostic accuracy verified by the receiver operator characteristic (ROC) analysis. Additionally, we constructed a nomogram that can effectively predict the OS rate of CRC patients. Furthermore, the signature determined that CRC cells from high-risk patients are more prone to recurrence and metastasis. Taken together, our ferroptosis-related lncRNA signature has a high prognostic value and may serve as a theoretical reference for personalized therapies for CRC patients. 

## 2. Materials and Methods

### 2.1. Data Collection and Preprocessing

We downloaded the RNA sequencing (RNA-seq) data, simple nucleotide variations data, and corresponding clinical data of CRC patients from The Cancer Genome Atlas (TCGA) database (https://portal.gdc.cancer.gov, accessed on 25 March 2021). The disease-specific survival (DSS) and progression-free interval (PFI) information of these patients were downloaded from the University of California, Santa Cruz (UCSC) Xena database (http://xena.ucsc.edu, accessed on 16 August 2021). Additionally, the progression-free survival (PFS) and disease-free survival (DFS) information were collected from the cBio Cancer Genomics Portal (cBioPortal) database (http://www.cbioportal.org, accessed on 16 August 2021). The protein expression data of CRC patients were obtained from the Clinical Proteomic Tumor Analysis Consortium (CPTAC) database (https://cptac-data-portal.georgetown.edu/study-summary/S016, accessed on 14 July 2021). Then, the expression levels of mRNAs and lncRNAs were converted to the transcripts per kilobase of exon signature per million mapped reads (TPM). The 505 patients with OS ≥ 30 days were retained for assuring the accuracy of the result in our analysis.

### 2.2. Identification of Differentially Expressed LncRNAs Related to Ferroptosis

The R package DESeq2 [35] was used for detecting the differentially expressed genes between normal tissues and CRC tissues. For further analysis, we filtered the genes that are not expressed in at least 20% of all samples to remove low-expressed genes. Besides, we downloaded 201 genes related to ferroptosis from the FerrDb database (http://www.zhounan.org/ferrdb, accessed on 12 March 2021) [36]. The differentially expressed lncRNAs whose expression levels were correlated with at least one of the differential ferroptosis-related genes (|R| > 0.5 and *p* < 0.001) were identified as the ferroptosis-related lncRNAs through the Pearson correlation analysis.

### 2.3. Establishment of the Prognostic Signature Composed of Ferroptosis-Related LncRNAs in the Training Set

The 505 screened CRC patients were regarded as an entire set and the R package caret [37] was used to randomly divide the entire set into a training set and a test set. The Fisher’s exact test was used to detect the differences in traditional clinical characteristics of the patients between the two sets. Then, we used the R package survival [38] to perform the univariate Cox regression analysis of OS for ferroptosis-related lncRNAs in the training set, and the prognostic lncRNAs were identified according to the criteria of *p* < 0.01. Additionally, to prevent the signature from overfitting, Least Absolute Shrinkage and Selection Operator (LASSO) Cox regression analysis was performed on the prognosis-related lncRNAs through the R package glmnet [39]. The stepwise multivariate Cox regression analysis was performed to construct the ferroptosis-related lncRNA prognostic signature by the smallest Akaike information criterion (AIC) value. Subsequently, the risk score of each patient was calculated by the following formula: risk score = $\overset{n}{\underset{i=1}{\sum }}\left({\mathrm{Coef}}_{i}*{\mathrm{Expr}}_{i}\right).$ The “Coef” represents the coefficient of each lncRNA in the results of multivariate Cox regression analysis, and “Expr” represents the expression level of each lncRNA.

### 2.4. Evaluation and Validation of the Prognostic Capability for the Prognostic Signature in the Test and Entire Sets

According to the optimal cut-point of the risk score of the training set determined by the surv_cutpoint function in survminer package (v.0.4.8, https://rpkgs.datanovia.com/survminer/index.html, accessed on 6 November 2020), the training, test, and entire set were divided into the high-risk and low-risk groups, respectively. Then, the Kaplan–Meier survival analysis and log-rank test were utilized to evaluate the differences in OS, RFS, DFS, DSS, PFS, and PFI of CRC patients between the high- and low-risk groups. Next, we used the R package survivalROC (v.1.0.3, https://cran.r-project.org/web/packages/survivalROC/index.html, accessed on 22 December 2020) to draw the time-dependent ROC curves of OS and evaluate the prediction accuracy of the prognostic signature based on the area under the curve (AUC) value. Subsequently, the risk curves for the two groups of CRC patients, as well as their overall survival time and survival status distribution diagram, were drawn to illustrate the relationship between the risk score and survival of patients. The expression heatmap of the eight lncRNAs in the signature was drawn by the R package heatmap.

### 2.5. The Relationship between Risk Score and Clinical Characteristics

According to various clinical characteristics, we divided the entire set into subgroups for analyzing the relationship between risk score and clinical characteristics, such as age (<65 years old, ≥65 years old), sex (female, male), TNM stage (stage I–II, stage III–IV), T stage (T1–2, T3–4), N stage (N0, N1–2), M stage (M0, M1), recurrence status (non-recurrence, recurrence), and survival status (alive, dead). Subsequently, the Fisher’s exact test was used to detect the differences in traditional clinical characteristics of the patients between the high- and low-risk groups. Moreover, the Wilcoxon test was used to calculate the difference in risk scores between different clinical subgroups. Additionally, the differences in OS between the high-risk group and low-risk group of these subgroups were analyzed by the Kaplan–Meier method and log-rank test.

### 2.6. Independent Prognostic Analysis of Risk Score and Construction of a Nomogram

To confirm whether risk score can be used as an independent prognostic indicator for CRC patients, we performed univariate and multivariate Cox analyses, together with the ROC curve analysis, based on risk score and clinical characteristics (age, sex, TNM stage, T stage, N stage, and M stage) in the training, test, and entire sets. Furthermore, based on the risk score and clinical characteristics (age, sex, and TNM stage) in the training set, we constructed a nomogram through the R package rms [40] to predict the OS rate of CRC patients and calculated the concordance index (C index) of the nomogram. Then, the bootstrap method was used for internal verification, and calibration curves of 1, 3, and 5 years were plotted to assess whether the survival rate predicted by the nomogram is consistent with the actual survival rate. Moreover, external verification for the prognostic value of the nomogram was also performed in the test and entire sets.

### 2.7. Functional Enrichment Analysis

Because the CRC patients’ proteomics data we downloaded in the CPTAC database are from TCGA patients, the protein expression data of the 70 patients with the risk scores in the CRC proteomics data are used as the proteomics set. We first compared the difference in OS between the high- and low-risk groups of CRC patients from the proteomics set by the Kaplan–Meier method and log-rank test. Then, the DESeq2 package was used to calculate the differential expression of genes between the high- and low-risk groups in the entire set based on the RNA-seq data, as well as the differential expression of proteins between the high- and low-risk groups in the proteomics dataset. The differentially expressed genes and proteins were used for the Kyoto Encyclopedia of Genes and Genomes (KEGG) enrichment analysis by R package ClusterProfiler [41]. The pathways with *p*.adjust < 0.05 were considered to be significantly enriched. Furthermore, all the genes of the entire set or all the proteins of the proteomics set were used to perform gene set enrichment analysis (GSEA) based on the KEGG pathway by the gseKEGG function of the ClusterProfiler package. The pathways with normalized enrichment score |NES| > 1 and *p*.adjust < 0.05 were considered to be significantly different. A positive value of NES indicates the pathway is enriched in the high-risk group, but a negative value indicates the pathway is enriched in the low-risk group. The epithelial–mesenchymal transition (EMT) score of each patient was calculated via the mean expression of the mesenchymal genes subtracted by the mean expression of the epithelial genes [42], and a higher EMT score implies that the cancer cell is closer to the mesenchymal phenotype. The difference in EMT scores between the high- and low-risk groups was calculated via the Wilcoxon test.

### 2.8. Analysis of Somatic Mutation Data

We analyzed and summarized the somatic mutation data of CRC patients using the R package maftools [43]. Then the Fisher’s exact test was performed to detect the genes whose mutation frequencies were different between the high- and low-risk groups. Subsequently, all the mutated-gene genes were used to perform GSEA analysis. Pathways with |NES| > 1 and *p* < 0.05 were considered to be significantly different.

### 2.9. Statistical Analysis

All statistical analyses were performed using R software (v.4.0.2, https://www.r-project.org, accessed on 22 June 2020). We used Fisher’s exact test to check the differences in categorical variables in different groups. DEseq2 was used to identify the differentially expressed genes and proteins between the two groups. The Pearson method was used to analyze the correlation between differential ferroptosis-related genes and differentially expressed lncRNAs. The differences in risk scores and EMT scores between different groups were calculated with the Wilcoxon test. The results with *p* < 0.05 were indicated as significant unless otherwise stated.

## 3. Results

### 3.1. Identification of Differentially Expressed Ferroptosis-Related LncRNAs in CRC Patients

**Figure 1:**
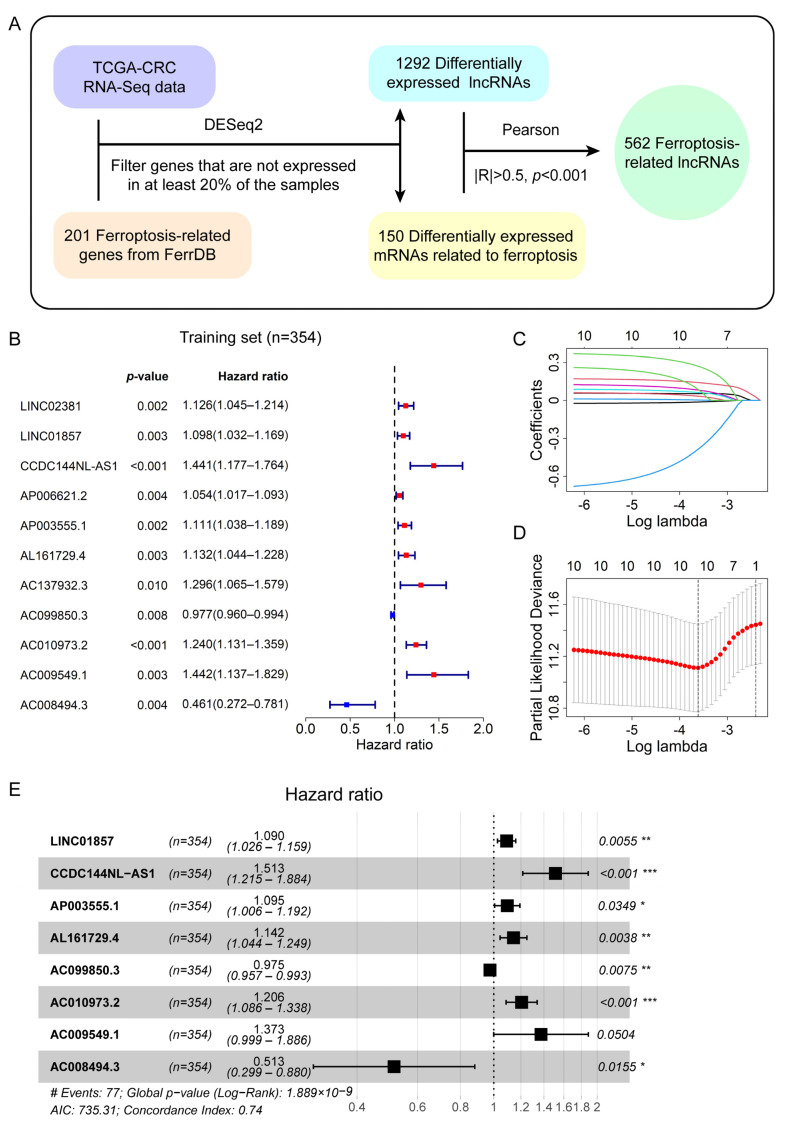
Construction of a prognostic signature composed of eight ferroptosis-related lncRNAs based on the training set. (**A**) Flowchart for the identification of ferroptosis-related lncRNAs. (**B**) The forest plot shows the 11 ferroptosis-related lncRNAs associated with OS identified by univariate Cox regression analysis. (**C**) The LASSO coefficient profile of the 10 OS-related lncRNAs displays the trajectory of the coefficients of the independent variable changing with the lambda. The upper abscissa represents the number of the independent variables with non-zero coefficients. (**D**) The cross-validation diagram shows that 10 candidate lncRNAs were screened out by LASSO regression analysis based on minimal error. The upper abscissa represents the number of independent variables. The vertical dashed line on the left was drawn according to the minimum criterion, corresponding to the optimal model. The vertical dashed line on the right was drawn according to one standard error of the minimum criteria (the 1-se criteria), corresponding to the signature with the fewest variables. (**E**) The forest plot shows a ferroptosis-related lncRNA prognostic signature constructed by multivariate Cox regression analysis. Unadjusted hazard ratios represent the 95% confidence intervals; * *p* < 0.05; ** *p* < 0.01; *** *p* < 0.001.

The flowchart for identifying ferroptosis-related lncRNAs in CRC patients is shown in Figure 1A. We firstly screened 4098 differentially expressed lncRNAs with the criteria of |log_2_(Fold Change)| > 1 and *p*.adjust < 0.05 between the normal and CRC tissues. Additionally, we downloaded 201 human genes related to ferroptosis from the FerrDb database and they are listed in Table S1. Then, we identified 155 differentially expressed ferroptosis-related mRNAs according to the standard of *p*.adjust < 0.05. Next, we removed the genes that were not expressed in 20% or more of all samples. As a result, 1292 differentially expressed lncRNAs and 150 ferroptosis-related mRNAs remained for further analysis. Then, the Pearson method was used to analyze the correlations between the expression levels of these lncRNAs and mRNAs. Subsequently, 562 lncRNAs, which we named ferroptosis-related lncRNAs, were screened out according to the threshold of correlation coefficient |R| > 0.5 and *p* < 0.001.

### 3.2. A Prognostic Signature Consisting of Eight Ferroptosis-Related LncRNAs Was Derived from the Training Set

The entire set obtained after filtration was randomly divided into a training set (*n* = 354) and a test set (*n* = 151) according to a ratio of 7:3 [44]. The clinical characteristics of the CRC patients in the training and test sets are shown in Table S2 and Fisher’s exact test showed there were no significant differences in these clinical characteristics of the patients between the two sets (*p* > 0.05). The detailed clinical information of each patient is shown in Table S3. In the training set, we used univariate Cox analysis to identify ferroptosis-related lncRNAs associated with the OS of CRC patients, and 11 lncRNAs were retained based on a cut-off value of *p* < 0.01 (Figure 1B). LncRNAs with a hazard ratio (HR) > 1 are associated with a poor prognosis in CRC patients, whereas lncRNAs with a HR < 1 are associated with a favorable prognosis in CRC patients. They were then further filtered by LASSO regression analysis and 10 lncRNAs related to prognosis remained (Figure 1C,D). Subsequently, we performed the multivariate Cox analysis and established a prognostic signature comprising eight ferroptosis-related lncRNAs associated with prognosis (Figure 1E, Table 1). Next, the risk score of each patient was calculated based on the coefficients (Table 1) and expression levels of these eight lncRNAs. The relationships of the eight lncRNAs of the prognostic signature and 20 ferroptosis-related mRNAs that are associated with their expression (|R| > 0.5, *p* < 0.001) were visualized in the mRNA–lncRNA co-expression network by Cytoscape software (v.3.8.2) [45] (Figure S1A). Correlations between these mRNAs and lncRNAs, as well as risk types, are also shown in the Sankey plot by the R software package ggalluval [46] (Figure S1B).

### 3.3. Evaluation and Validation of the Prognostic Performance for the Prognostic Signature

**Figure 2:**
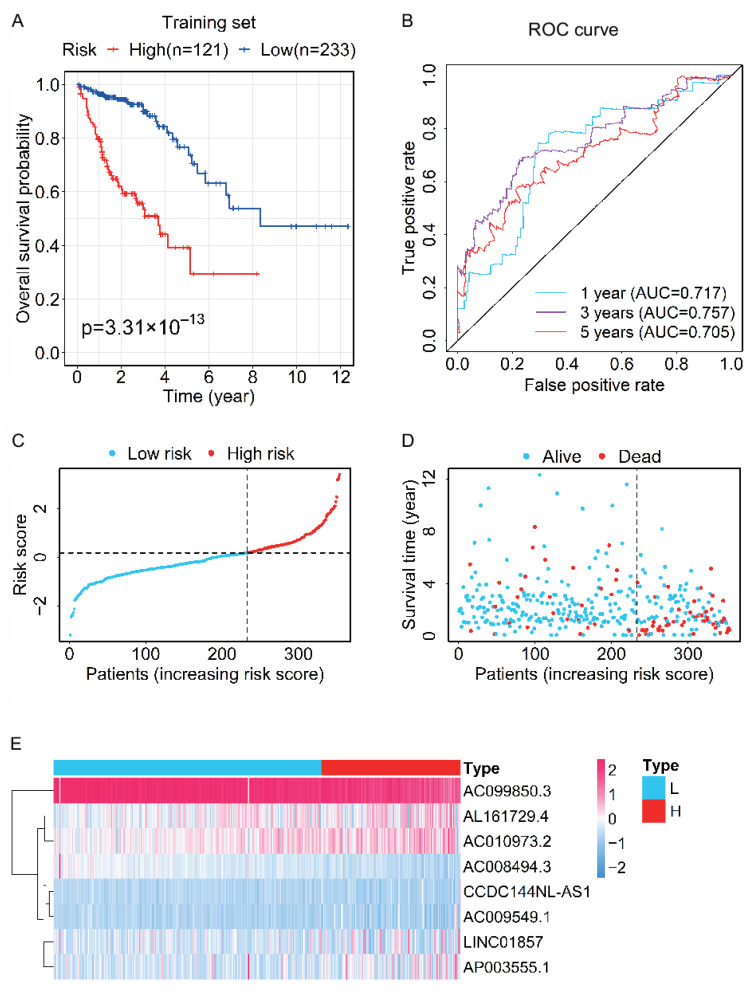
Evaluation for the prognostic value of the lncRNA signature in the training set. (**A**) Kaplan–Meier survival curve for OS of CRC patients in the high- and low-risk groups. (**B**) The time-dependent ROC curves of OS based on the risk score indicate the prognostic accuracy of the 8-lncRNAs signature. (**C**) The distribution of risk scores of CRC patients. (**D**) The scatter plot shows the overall survival time and survival status of CRC patients in the high- and low-risk groups. (**E**) Heatmap of clustering analysis for the expression of eight ferroptosis-related lncRNAs. L, low risk; H, high risk.

The training set was divided into a high-risk group (*n* = 121) and a low-risk group (*n* = 233) via the optimal cut-point of the risk score (risk score = 0.1826). Furthermore, the Kaplan–Meier curve showed the difference in OS between the high- and low-risk groups in the training set (Figure 2A). The OS of patients in the high-risk group was significantly shorter than that in the low-risk group, suggesting this signature was valuable for the prognosis of CRC. Furthermore, the AUC values at one, three, and five years of the ROC curve for the risk score were 0.717, 0.757, and 0.705, respectively, indicating the signature had good prognostic accuracy (Figure 2B). The risk curve, together with the overall survival time and survival status distribution graph, showed the OS of patients was associated with the risk score (Figure 2C,D). The overall survival time in the high-risk group was lower than that in the low-risk group, and the number of deaths in the high-risk group was also higher. The heatmap of clustering analysis for the expression levels of eight ferroptosis-related lncRNAs displayed that AL161729.4, AC010973.2, CCDC144NL-AS1, AC009549.1, LINC01857, and AP003555.1 were highly expressed in the high-risk group, while AC099850.3 and AC008494.3 were highly expressed in the low-risk group (Figure 2E).

**Figure 3:**
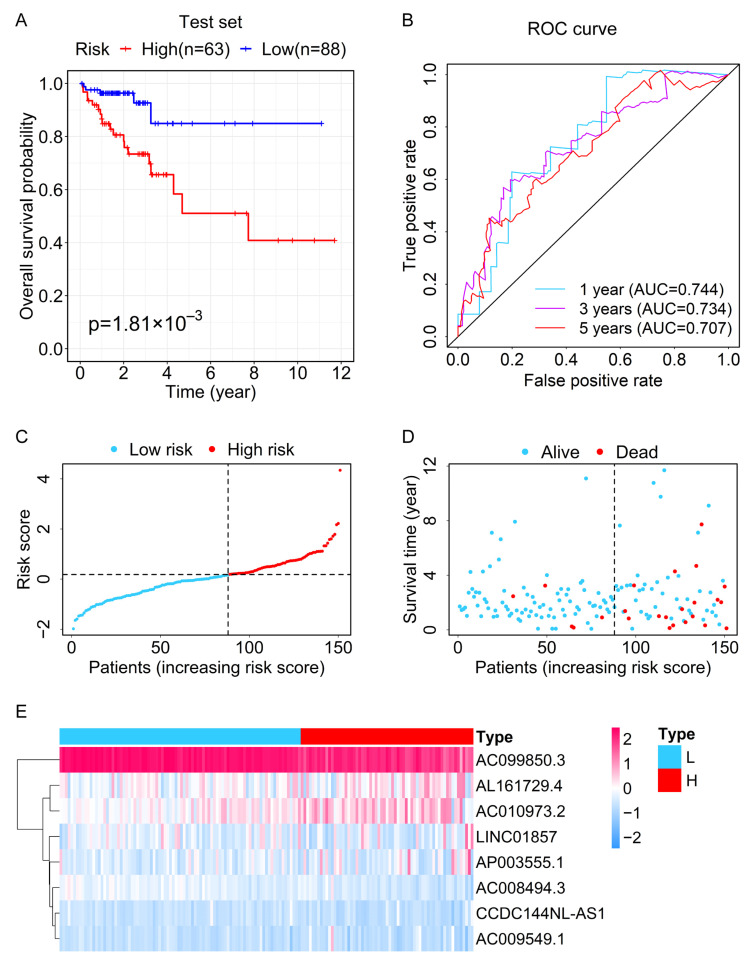
Validation for the prognostic value of the lncRNA signature in the test set (**A**) Kaplan–Meier survival curve for OS of CRC patients in the high- and low-risk groups. (**B**) The time-dependent ROC curves of OS based on the risk score indicate the prognostic accuracy of the 8-lncRNAs signature. (**C**) The distribution of risk scores of CRC patients. (**D**) The scatter plot shows the overall survival time and survival status of CRC patients in the high- and low-risk groups. (**E**) Heatmap of clustering analysis for the expression of eight ferroptosis-related lncRNAs in the high- and low-risk groups. L, low risk; H, high risk.

We used the same formula as the training set to calculate the risk score of each patient in the test set. According to the optimal cut-point of the risk score in the training set (risk score = 0.1826), the test set was also categorized into the high-risk group (*n* = 63) and low-risk group (*n* = 88). The Kaplan–Meier survival curve showed that patients in the high-risk group had a lower OS rate and a poorer prognosis than those in the low-risk group (Figure 3A). Moreover, the ROC analysis showed that this prognostic signature had stable prognostic ability for CRC patients (one-year AUC = 0.744, three-year AUC = 0.734, five-year AUC = 0.707; Figure 3B). The risk curve and survival graphs are displayed in Figure 3C,D. The heatmap of clustering analysis for the expression levels of eight ferroptosis-related lncRNAs in the high-risk and low-risk groups of the test set is shown in Figure 3E.

The same method was used to calculate the risk score of each patient in the entire set, which was classified into a high-risk group (*n* = 184) and a low-risk group (*n* = 321) based on the optimal cut-point of the risk score derived from the training set (risk score = 0.1826). Consistent with previous results, patients in the high-risk group had a worse prognosis in OS (Figure S2A). At one, three, and five years, the AUC values were 0.724, 0.745, and 0.711, respectively (Figure S2B). The risk curve and survival status plots indicated that a higher patient risk score means a higher mortality rate (Figure S2C,D). The heatmap of clustering analysis for the expression levels of the eight ferroptosis-related lncRNAs is shown in Figure S2E.

To further evaluate the more common clinical indicators related to prognosis, we compared the RFS, DFS, DSS, PFS, and PFI rates of CRC patients. As observed in the training, test, and entire sets, all these indicators of patients in the high-risk group were also lower than those in the low-risk groups in the test or entire sets (Figures S3 and S4). All the above results indicated that the ferroptosis-related lncRNA signature could be used as a valuable prognostic indicator for CRC patients.

### 3.4. Analysis of the Relationship between the Prognostic Signature and the Clinical Characteristics of CRC Patients

**Figure 4:**
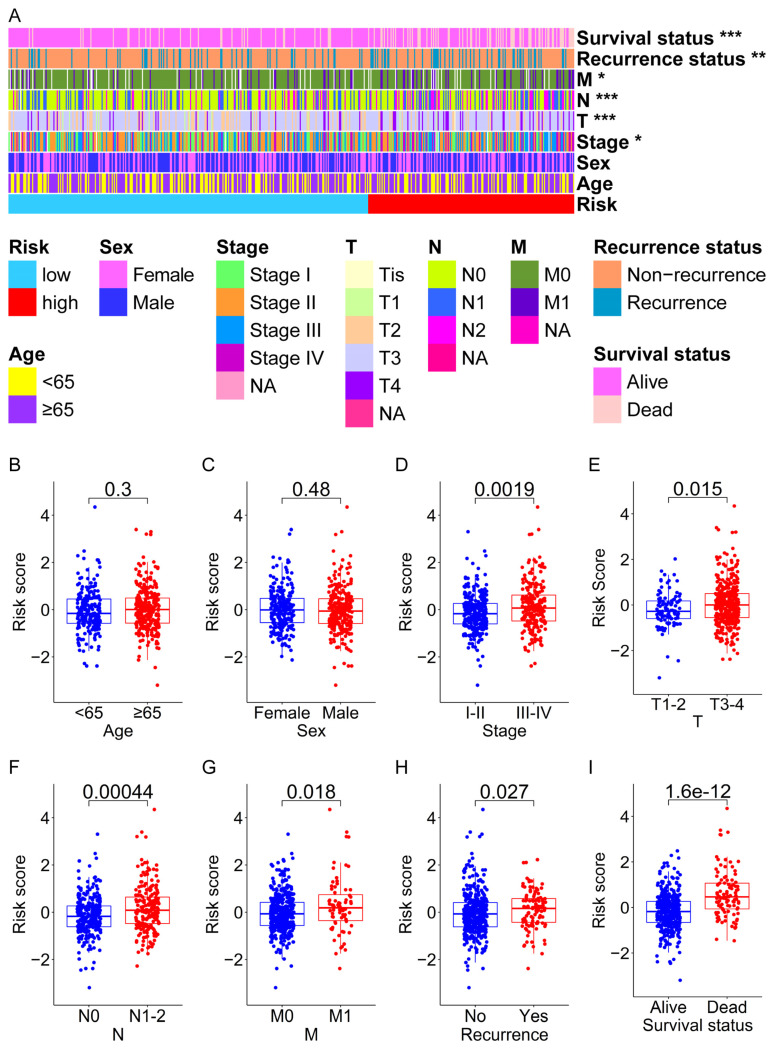
The relationship between the risk score and clinical characteristics of CRC patients in the entire set. (**A**) The heatmap shows the distribution of clinical characteristics of patients between high-risk and low-risk groups. The box plots show that there is no significant difference in risk scores between patients with different ages (≥65 years old, <65 years old) (**B**) or patients with different sexes (female, male). (**C**,**D**) The box plot shows the risk scores of CRC patients with stage III–IV are significantly higher than that of stage I–II. (**E**) The box plot shows that the risk scores of CRC patients with the T3–4 stage are significantly higher than those of T1–2. (**F**) The box plot shows that the risk scores of CRC patients with the N1–2 stage are significantly higher than those of N0. (**G**) The box plot shows that the risk scores of CRC patients with the M1 stage are significantly higher than those of M0. (**H**) The box plot shows that the risk scores of CRC patients with tumor recurrence are significantly higher than those of tumor non-recurrence. (**I**) The box plot shows that the risk scores of dead patients are significantly higher than those of alive patients. * *p* < 0.05; ** *p* < 0.01; *** *p* < 0.001.

The Fisher’s exact test revealed that there were no significant differences in the age or gender of the patients between the high- and low-risk groups, but there were significant differences in TNM stage, T stage, N stage, M stage, recurrence status, and survival status of the patients between the high- and low-risk groups (Table S4 and Figure 4A). To explore further whether the risk score is related to the clinical characteristics of CRC patients, we divided the patients in the entire set into two subgroups according to age (≥65 years old, <65 years old), sex (female, male), TNM stage (stage I-II, stage III-IV), T stage (T1–2, T3–4), N stage (N0, N1–2), and M stage (M0, M1), and determined whether there was a difference in risk scores between the two subgroups. As shown in Figure 4B,C, no correlation existed between the risk score and age or sex (*p* > 0.05). However, the risk score was related to the TNM stage, T stage, N stage, M stages, recurrence status, and survival status because there were statistical differences between the risk scores of their two subgroups (*p* < 0.05; Figure 4D–I). Moreover, CRC patients with higher pathological stages had higher risk scores.

**Figure 5:**
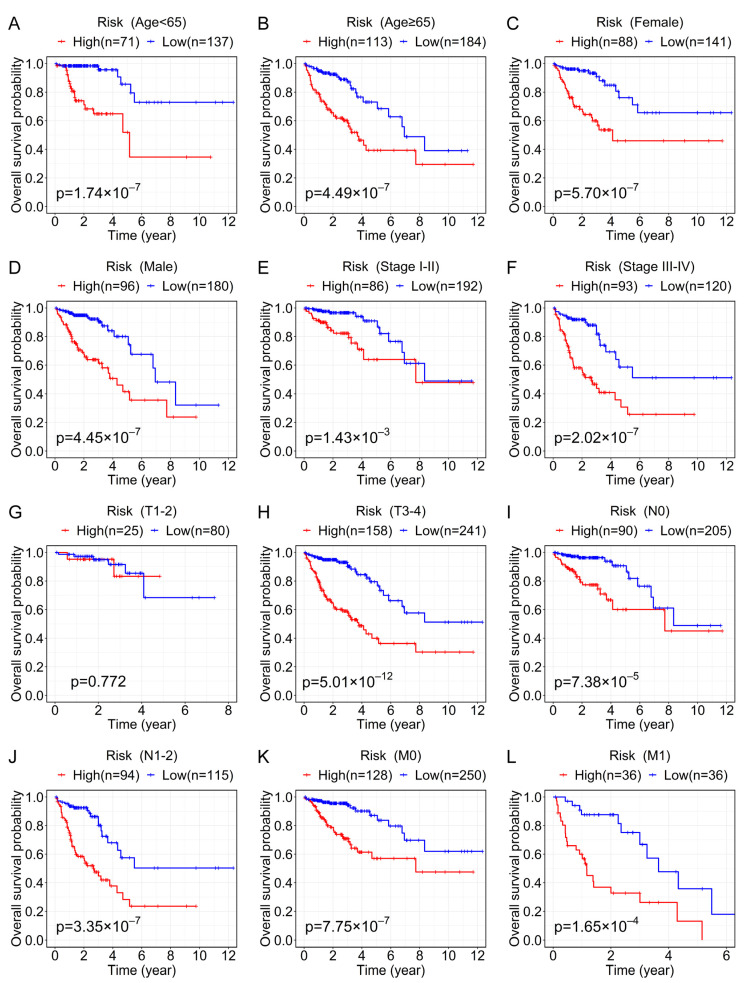
Prognostic value of the prognostic signature for CRC patients with different clinical characteristics. Kaplan–Meier survival curve analysis for OS shows the differences between the high- and low-risk groups in multiple clinical subgroups of CRC patients in the entire set, including age < 65 years old (**A**), age ≥ 65 years old (**B**), female (**C**), male (**D**), stage I–II (**E**), stage III–IV (**F**), T1–2 (**G**), T3–4 (**H**), N0 (**I**), N1–2 (**J**), M0 (**K**), and M1 (**L**).

Furthermore, to determine the prognostic value of the prognostic signature in various clinical features, we used the Kaplan–Meier method and log-rank test to perform survival analysis between the high- and low-risk groups in each subgroup. As shown in Figure 5, except for stage T1–2 (*p* > 0.05; Figure 5G), the prognosis of patients with high-risk scores was worse than that of patients with low-risk scores in other subgroups (*p* < 0.05; Figure 5A–F,H–L). These findings indicated that the prognostic signature was applicable to a large number of patients. Additionally, the higher the risk score is at the same pathological stage, the worse the patient’s prognosis may be.

### 3.5. The Prognostic Signature Can Be Applied as an Independent Prognostic Factor for CRC Patients

**Figure 6:**
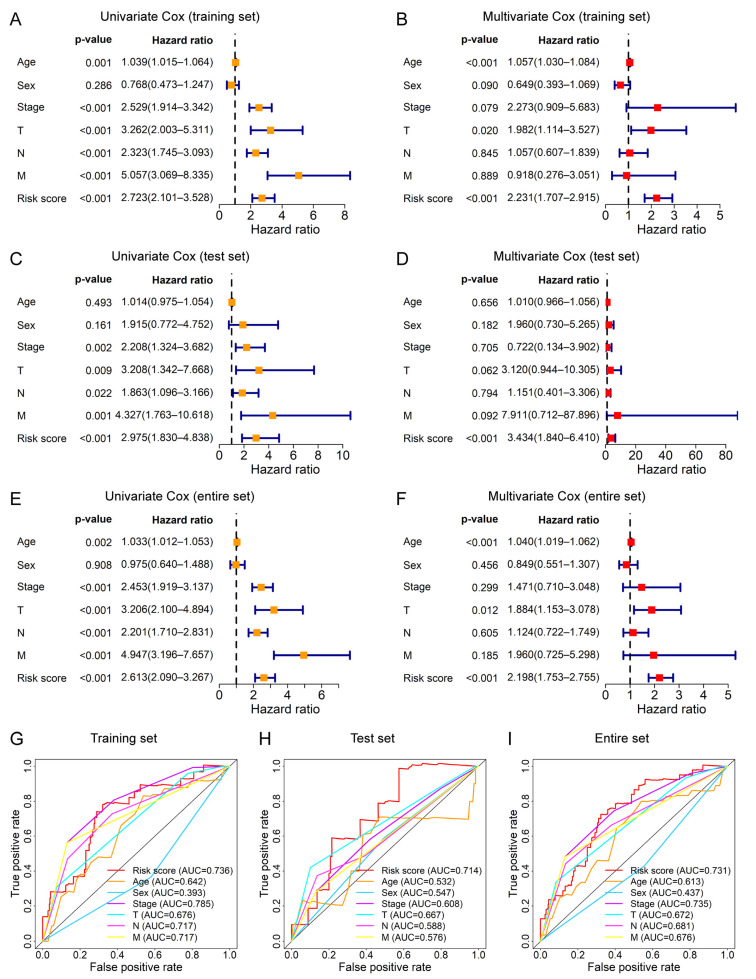
Assessment for the prognostic independence of the risk score. (**A**,**B**) show the univariate Cox and multivariate Cox analysis of OS based on the risk score and clinical characteristics (age, sex, TNM stage, T stage, N stage, and M stage) in the training set, respectively. (**C**,**D**) show the univariate Cox and multivariate Cox analysis of OS based on the risk score and clinical characteristics in the test set, respectively. (**E**,**F**) show the univariate Cox and multivariate Cox analysis of OS based on the risk score and clinical characteristics in the entire set, respectively. The 1-year ROC curves of OS based on the risk score and clinical characteristics in the training set (**G**), test set (**H**), and entire set (**I**) indicate the prognostic accuracy of these variables. Unadjusted hazard ratios represent the 95% confidence intervals.

Univariate and multivariate Cox analyses were employed to determine whether the ferroptosis-related lncRNA signature can be used as an independent prognostic factor for CRC patients and the results are shown in the forest plot (Figure 6). In the training set, the univariate Cox analysis showed that the risk score of the ferroptosis-related lncRNA signature was correlated with the prognosis of CRC patients (Figure 6A) and the multivariate Cox analysis showed that the risk score can be taken as an independent prognostic factor for CRC patients (Figure 6B).

In the test set, univariate Cox analysis (Figure 6C) indicated that risk score was related to prognosis, and multivariate Cox analysis (Figure 6D) also proved that risk score was an independent prognostic factor. Similarly, in the entire set, univariate Cox analysis (Figure 6E) and multivariate Cox analysis (Figure 6F) also showed that the risk score can be used as an independent prognostic factor. Moreover, the AUC values of the risk score were also higher than those of the majority of clinical characteristics, including age, sex, T stage, N stage, and M stage, in the training, test, and entire sets (Figure 6G–I). Taken together, the risk score of the ferroptosis-related lncRNA signature was a significant independent prognostic factor for CRC patients.

### 3.6. Construction and Verification of a Nomogram for the Prediction of the OS Rate

**Figure 7:**
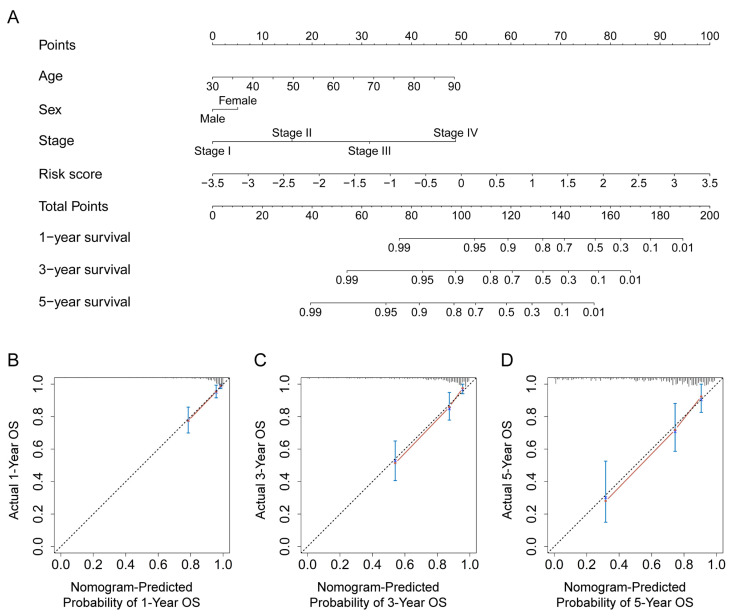
Construction and verification of the nomogram in the training set. (**A**) The established nomogram, which contains several indicators, such as age, sex, TNM stage, and risk score, can predict the OS rate of 1, 3, and 5 years for CRC patients in the training set. The calibration curves at 1-year (**B**), 3-year (**C**), and 5-year (**D**) OS of the nomogram in the training set show the predicted result of the nomogram is close to the actual OS rate.

To predict the OS rate, we constructed a nomogram in the training set using various indicators, including the risk score and clinical characteristics. The total point calculated by summing the scores of the indicators can predict OS rates of 1, 3, and 5 years for CRC patients (Figure 7A). The derived C-index of 0.820 (95% CI = 0.767–0.873, *p* < 0.001) indicated that the nomogram had an accurate predictive ability. Furthermore, the calibration curves of 1, 3, and 5 years showed that the OS rates predicted by the nomogram were well consistent with the actual OS rates (Figure 7B–D).

Additionally, the C-index values of the nomogram in the test and entire sets were 0.727 (95% CI = 0.601–0.853, *p* < 0.001) and 0.796 (95% CI = 0.744–0.847, *p* < 0.001), respectively, indicating the nomogram derived from the training set also had a stable predictive power in both the test and entire sets. Furthermore, the accuracy of the OS rate generated by the nomogram was validated by the 1-, 3-, and 5-year calibration curves in the test and entire sets (Figure S5). As a result, the nomogram had a reliable predictive performance.

### 3.7. Functional Enrichment Analysis Revealed the Biological Processes Related to the Prognostic Signature

**Figure 8:**
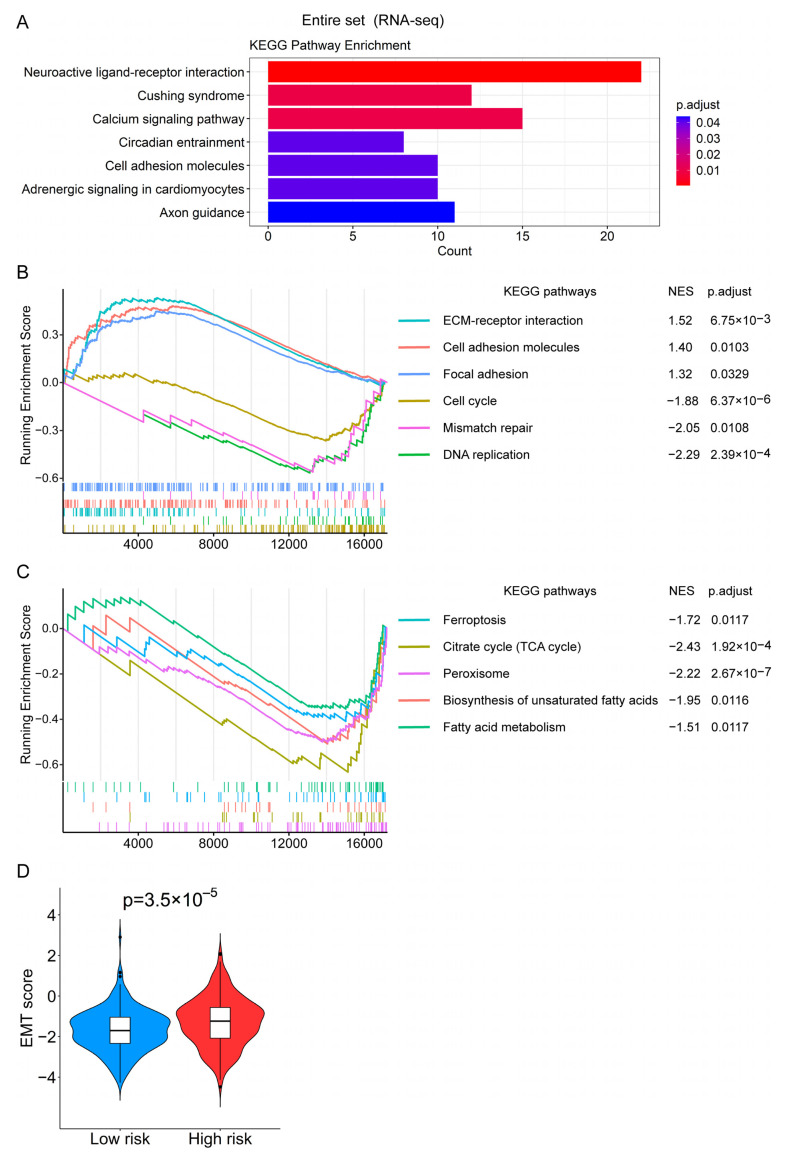
The possible biological processes related to the risk score revealed by the functional enrichment analysis. (**A**) The significantly enriched KEGG pathways of the differentially expressed genes between the high- and low-risk groups in the entire set. (**B**) The significantly enriched KEGG pathways between the high- and low-risk groups in the entire set based on the RNA-seq data through the GSEA analysis. (**C**) The “Ferroptosis” pathway and some metabolic pathways related to it are enriched in the low-risk group of the entire set based on the RNA-seq data via the GSEA analysis. (**D**) The violin plot shows the difference in EMT scores between the high- and low-risk groups in the entire set.

To find the factors that may lead to the significant difference in OS between the high-risk group and the low-risk group, we explored some possible biological pathways and processes. The 462 differentially expressed genes between the high- and low-risk groups were identified with |log_2_ (fold change)| > 1 and *p*.adjust < 0.05. KEGG enrichment analysis showed that these differentially expressed genes were enriched in the “Cell adhesion molecules” pathway that related to tumor metastasis (Figure 8A). Moreover, GSEA analysis with all the genes revealed that “ECM–receptor interaction”, “Cell adhesion molecules”, and “Focal adhesion” pathways were enriched in the high-risk group, whereas “DNA replication”, “Mismatch repair”, and “Cell cycle” were enriched in the low-risk group (Figure 8B). Interestingly, the “ferroptosis” pathway and some metabolic pathways related to it, including “Citrate cycle (TCA cycle)”, “Peroxisome”, “Biosynthesis of unsaturated fatty acids”, and “Fatty acid metabolism”, were enriched in the low-risk group (Figure 8C). Furthermore, the EMT scores in the high-risk group were also significantly higher than those in the low-risk group, which indicated CRC cells in the high-risk group were more inclined to be the mesenchymal phenotype (Figure 8D).

As shown in Figure S6A, the OS of patients in the high-risk group was significantly shorter than that of the low-risk group in the proteomics dataset. Therefore, we explored the differences in expression levels of proteins between the high- and low-risk groups. We identified 320 differentially expressed proteins with the standard of *p* < 0.05 between the high- and low-risk groups. Subsequently, we found that the expression of Ki-67, a cell proliferation marker protein, was significantly down-regulated in the high-risk group (Figure S6B). These 320 differentially expressed proteins were primarily enriched in some metabolic pathways, together with several cancer-related pathways such as “DNA replication”, “ECM-receptor interaction”, “Mismatch repair”, and “Ferroptosis” pathways (Figure S6C). GSEA analysis with all the proteins also demonstrated that “ECM-receptor interaction” and “Focal adhesion” pathways were enriched in the high-risk group, whereas “DNA replication”, “Mismatch repair”, and “Cell cycle” pathways were enriched in the low-risk group (Figure S6D). Based on the above analysis, it can be assumed that cancer cells in the high-risk group may be related to the suppression of the cell cycle, metastasis, mutation, and the inhibition of ferroptosis.

### 3.8. The Gene Mutation Profiling of CRC Patients between the High- and Low-Risk Groups

**Figure 9:**
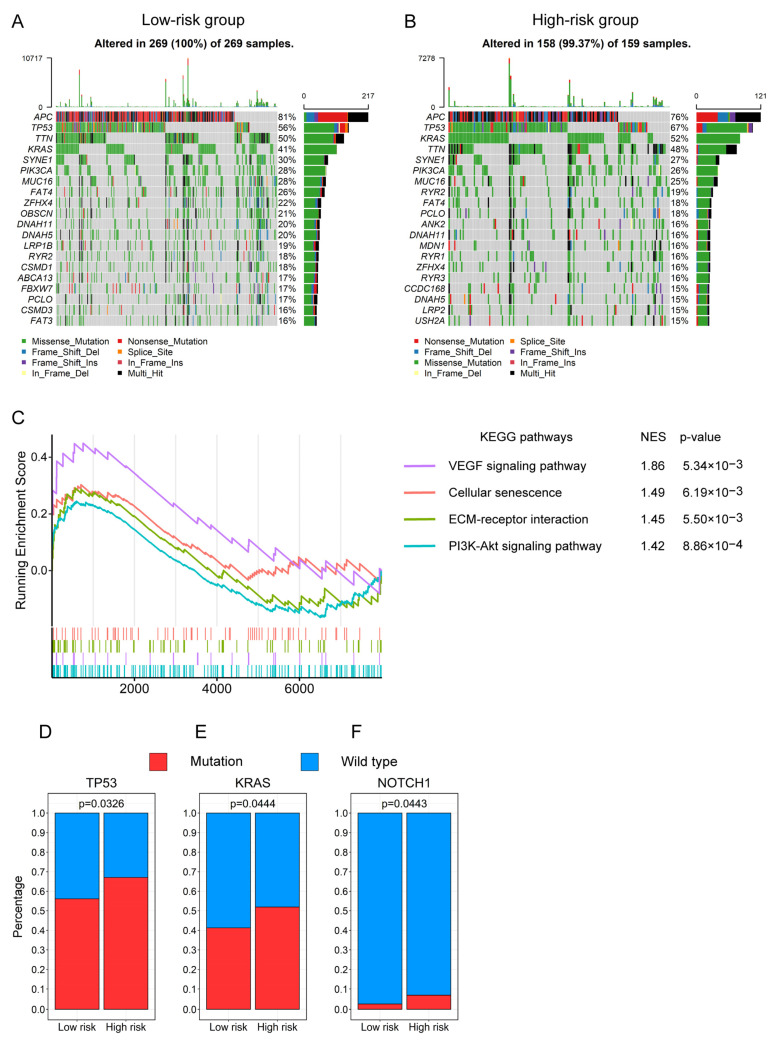
The somatic mutation landscape of CRC patients between the high- and low-risk groups. Waterfall plots display the distribution of variant classifications of the top 20 genes with higher mutation frequencies in the low-risk group (**A**) and high-risk group (**B**). (**C**) The significantly enriched KEGG pathways between the high- and low-risk groups in the somatic mutation data through the GSEA. Bar plots show the different mutation frequencies of *TP53* (**D**), *KRAS* (**E**), and *NOTCH1* (**F**) genes between the high- and low-risk groups in the somatic mutation data.

As the “Mismatch repair” pathway related to mutation was significantly enriched in the low-risk groups, we further analyzed and summarized the somatic mutation data of CRC patients in the high- and low-risk groups. The top 20 genes sorted by alteration frequency in the high- or low-risk groups are shown in the waterfall plots (Figure 9A,B). Then, we compared the difference in the mutation frequencies of genes between the high- and low-risk groups for uncovering the possible pathways involved in the gene mutation heterogeneity between the two groups. Subsequently, we found that the “VEGF signaling pathway”, “Cellular senescence”, “ECM–receptor interaction”, and “PI3K-Akt signaling pathway” were significantly enriched in the high-risk group, according to the result of the GSEA analysis (Figure 9C). Additionally, the mutation frequencies of *TP53* (67.09% vs. 56.13%), *KRAS* (51.90% vs. 41.26%), and *NOTCH1* (6.96% vs. 2.60%) genes were significantly increased in the high-risk group compared to the low-risk group (Figure 9D–F). These results implied that the high-risk score was associated with the gene mutation.

Generally, the mutants of *TP53* and *KRAS* genes play oncogenic roles in the tumorigenesis and development of CRC [47,48]. One previous study showed that *TP53* mutations are prevalent in metastatic CRC compared to the primary tumor [49]. Besides, the specific *TP53* or *KRAS* mutations are associated with poor survival in CRC [50,51], and the double mutation of *TP53* and *RAS* is related to a worse prognosis for patients after colorectal liver metastases resection [52]. Similarly, the mutated *NOTCH1* could cause abnormal activation in the Notch signaling pathway, which contributes to CRC behaviors [53]. Notably, the *NOTCH1* mutation is the most frequently occurring somatic mutation in recurrent CRC samples [54]. Hence, our findings that the high-risk group has a higher mutation frequency of *TP53*, *KRAS*, and *NOTCH1* may help to explain why this group has a worse prognosis.

## 4. Discussion

Increasing studies reported that lncRNA expression can be modulated by ferroptosis [29,30,31], a biological process that may play a tumor-suppressive role in CRC progression [19,20,21,22]. In this article, we combined multiple ferroptosis-related lncRNAs as a prognostic factor, which generally possesses higher accuracy and reliability than a single prognostic factor [5,6]. Firstly, we identified 562 ferroptosis-related lncRNAs based on the RNA-seq data of CRC from the TCGA database. Then, eight of them were selected to establish an optimal prognostic signature, which showed a stable ability to predict the prognosis of CRC patients. The prognostic signature can judge that the OS of CRC patients in the high-risk group was significantly shorter than that of patients in the low-risk group. Moreover, it showed broadly applicable performance in different subgroups. In addition to OS, the lncRNA signature can also predict the RFS, DFS, DSS, PFS, and PFI of CRC patients. Furthermore, the nomogram based on the risk score, age, sex, and TNM stage can accurately predict the OS rates of CRC patients.

For the eight ferroptosis-related lncRNAs constructing the prognostic signature, two lncRNAs (AC008494.3 and AC099850.3) are protective factors and the other six lncRNAs (AC009549.1, AC010973.2, AL161729.4, AP003555.1, CCDC144NL-AS1, and LINC01857) are risk factors. One recent study based on bioinformatics analysis reported that AC010973.2 and AL161729.4 are prognostic risk factors in CRC, while AC099850.3 is a protective factor [55]. Additionally, CCDC144NL-AS1 and LINC01857 have been experimentally studied in cancers. CCDC144NL-AS1 can increase cell proliferation, migration, and invasion of gastric cancer and is associated with a poor prognosis in gastric cancer patients [56]. Similarly, LINC01857 has been revealed to promote progression in cancers, such as glioma [57], B-cell lymphoma [58], and breast cancer, and predict the poor prognosis of breast cancer patients [59]. Therefore, all the above previous studies supported the lncRNAs in our prognostic signature function as protective or risk factors in CRC.

Although the TNM stage is currently the most widely used prognostic indicator for CRC patients, it is not without inherent limitations, including a lack of prediction accuracy [6], insufficient prognostic information [60], and significant clinical outcome disparities among CRC patients with the same histological tumor stage [61]. Therefore, it is urgently needed to find new prognostic factors as the supplement to the TNM staging system for the prognosis and treatment of CRC. Based on some clinical characteristics and risk scores, univariate and multivariate Cox regression analyses were performed in our study to prove that the ferroptosis-related lncRNA signature is an independent prognostic risk factor for CRC patients. Moreover, the ROC analysis indicated the prediction accuracy of the prognostic signature was better than most of the other clinical characteristics, including age, sex, T stage, N stage, and M stage. The validation of the above results in the test and entire sets further supported that the signature can be utilized as a reliable prognostic indicator for CRC patients.

To elucidate the mechanism underlying the difference in prognosis between high- and low-risk groups, we used gene enrichment analysis and GSEA to explore the biological processes that differentiate the two groups using multi-omics data. Our analysis showed that not only “Cell cycle” and “DNA replication” were de-enriched based on the RNA-seq and proteomics data (Figure 8B and Figure S6D), but also the protein expression level of Ki-67 was decreased in the high-risk group (Figure S6B), which indicated the CRC cells in this group might enter a cell cycle arrest, a status of dormancy [62]. Previous studies demonstrated that dormant tumor cells, including CRC cells, are responsible for the metastatic relapse of primary tumors [62,63,64]. Mechanistically, during dormancy, the tumor cells can acquire more mutations that are necessary for expanding neoplastic processes and preparing the metastatic dissemination followed by subsequent outgrowth [62], which leads to the greatest part of the morbidity and death of many solid tumors [65]. Consistently, using the somatic mutation data, we discovered some cancer processes, such as “PI3K-Akt signaling pathway”, “VEGF signaling pathway”, “ECM–receptor interaction”, and “Cellular senescence”, were significantly enriched in the high-risk group (Figure 9C), which verified more cancer-related mutations happen in the group with high risk.

Additionally, the “ECM–receptor interaction” and “Focal adhesion” pathways were enriched in the high-risk group based on the RNA-seq and proteomics data (Figure 8B; Figure S6D). Moreover, the CRC cells in the high-risk group may tend to be the mesenchymal phenotype (Figure 8D). These findings further implied CRC cells in this group may have higher metastatic potential. Interestingly, one recent study reported that “Focal adhesion” and “ECM–receptor interaction” are two of the enriched pathways for the proteins that were up-regulated in liver metastatic CRC tissues compared to primary CRC tissues [66], which also shows the enrichment of these pathways is connected with metastasis. Furthermore, the patients in the high-risk groups had lower RFS (Figure S3A–C) and DFS (Figure S3D–F), as well as a higher tumor recurrence rate (Figure 4A,H), which also confirmed the above results. Taken together, based on our analysis data, we postulated that the tumor cells of CRC patients in the high-risk group may have entered a dormant period and prepared for subsequent recurrence and metastasis.

Notably, we discovered that the differentially expressed proteins between the high- and low-risk groups were enriched in the “Ferroptosis” pathway. Moreover, the GSEA of the entire set showed that the “Ferroptosis” pathway was enriched in the low-risk group (Figure 8C). Previous studies revealed the inhibitory function of ferroptosis on the tumorigenesis and development of CRC [19,22], which may help to explain the phenomenon in our study that the low-risk group had a good prognosis. In addition to “Ferroptosis”, some metabolism-related biological processes interplaying with the ferroptosis pathway [9,10,11], including “Peroxisome”, “Citrate cycle (TCA cycle)”, “Fatty acid metabolism”, and “Biosynthesis of unsaturated fatty acids”, were also enriched in the low-risk group (Figure 8C). Some recent studies found that the “TCA cycle” can promote ferroptosis via the accumulation of lipid peroxide, thereby suppressing cancer progression [9,67,68]. Besides, as substrates for lipid peroxidation, polyunsaturated ether phospholipids synthesized from peroxisome [10,69,70] and polyunsaturated fatty acids synthesized by fatty acid synthase [11,68] also contribute to ferroptosis. Therefore, these biological pathways may synergistically enhance ferroptosis, resulting in the suppression of CRC occurrence and development of patients in the low-risk group.

In brief, we developed a ferroptosis-related lncRNA signature that was closely related to the prognosis of CRC patients and further verified its discriminative accuracy. Then, based on the risk score and other clinical characteristics, we constructed a nomogram that can effectively predict the OS rate of CRC patients. Nonetheless, our signature has certain limitations. Because we validated the signature only with internal data from the TCGA database, it will be more convincing to find suitable external data for verification. As the functions and functional mechanisms by which the eight lncRNAs of the signature participating in the ferroptosis in CRC are still unknown, further verification based on experimental evidence will be required. Although there are imperfections that need to be improved, our signature could provide new insights and a theoretical basis for the prognosis and treatment of CRC patients.

## 5. Conclusions

In summary, we established a novel prognostic signature with eight ferroptosis-related lncRNAs to evaluate the prognosis of CRC patients. Moreover, the lncRNA signature was also associated with the recurrence and metastasis of CRC. Apart from its broad applicability and accuracy, our signature also revealed several possible explanations for the poor prognosis of CRC patients in the high-risk group, such as tumor dormancy and anti-ferroptosis. Therefore, our study may provide a theoretical foundation for the investigation of the pathological mechanisms and clinical management strategies for CRC.

## Figures and Tables

**Table 1:** The coefficients of eight ferroptosis-related lncRNAs of the multivariable Cox regression analysis in the training set.

Gene Symbol	Ensembl ID	Genomic Location (GRCh38)	Coefficient
LINC01857	ENSG00000224137	Chr2: 207,662,375–207,667,024	0.0865
CCDC144NL-AS1	ENSG00000233098	Chr17: 20,868,433–21,002,276	0.4138
AP003555.1	ENSG00000254605	Chr11: 70,014,858–70,021,059	0.0909
AL161729.4	ENSG00000271659	Chr9: 95,514,045–95,514,520	0.1325
AC099850.3	ENSG00000265415	Chr17: 59,202,677–59,203,829	−0.0251
AC010973.2	ENSG00000244151	Chr7: 151,074,742–151,076,530	0.187
AC009549.1	ENSG00000270607	Chr11: 19,710,934–19,712,619	0.3169
AC008494.3	ENSG00000271797	Chr5: 115,262,505–115,263,448	−0.668

## Data Availability

The data analyzed in this study are available in the TCGA, CPTAC, and FerrDb databases.

## Supplementary Materials

The following supporting information can be downloaded at: https://www.mdpi.com/article/10.3390/genes13061094/s1. Table S1. 201 human genes related to ferroptosis download from the FerrDb database. Table S2. The clinical characteristics of colorectal cancer patients between the training and test sets. Table S3. The detailed clinical characteristics of colorectal cancer patients. Table S4. The clinical characteristics of patients between the high- and low-risk groups in the entire set. Figure S1. Co-expression network and Sankey relationship diagram between eight lncRNAs in the prognostic signature and their related mRNAs associated with ferroptosis. Figure S2. Verification for the prognostic value of the prognostic signature in the entire set. Figure S3. Evaluation and verification of the ferroptosis-related lncRNA prognostic signature for predicting RFS and DFS of CRC patients. Figure S4. Evaluation and verification of the ferroptosis-related lncRNA prognostic signature for predicting DSS, PFS, and PFI of CRC patients. Figure S5. The calibration curves for the nomogram derived from the training set. Figure S6. The functional enrichment analysis between the high- and low-risk groups in the proteomics dataset.
