# Supplementary material for: Integrated Analysis of a Ferroptosis-Related LncRNA Signature for Evaluating the Prognosis of Patients with Colorectal Cancer
Source: Genes (Basel). 2022 Jun 19;13(6):1094. doi: 10.3390/genes13061094 (PMC9223081; doi:10.3390/genes13061094)
Supplement: Supplementary file 1 [file genes-13-01094-s001.zip › genes-1731490-supplementary.pdf]

Supplementary Materials

Table S1. 201 human genes related to ferroptosis download from the FerrDb database.

Gene symbol	Biotype
RPL8	protein_coding
IREB2	protein_coding
ATP5MC3	protein_coding
CS	protein_coding
EMC2	protein_coding
ACSF2	protein_coding
NOX1	protein_coding
CYBB	protein_coding
NOX3	protein_coding
NOX4	protein_coding
NOX5	protein_coding
DUOX1	protein_coding
DUOX2	protein_coding
G6PD	protein_coding
PGD	protein_coding
VDAC2	protein_coding
TP53	protein_coding
ACSL4	protein_coding
LPCAT3	protein_coding
NRAS	protein_coding
KRAS	protein_coding
HRAS	protein_coding
CARS1	protein_coding
KEAP1	protein_coding
HMOX1	protein_coding
ATG5	protein_coding
ATG7	protein_coding
NCOA4	protein_coding
TF	protein_coding
ALOX5	protein_coding
ALOX12	protein_coding
ALOX12B	protein_coding
ALOX15	protein_coding
ALOX15B	protein_coding
ALOXE3	protein_coding
PHKG2	protein_coding
SAT1	protein_coding
EGFR	protein_coding
MAPK3	protein_coding

MAPK1	protein_coding
ZEB1	protein_coding
DPP4	protein_coding
CDKN2A	protein_coding
PEBP1	protein_coding
SOCS1	protein_coding
CDO1	protein_coding
MYB	protein_coding
SLC1A5	protein_coding
CHAC1	protein_coding
GOT1	protein_coding
BECN1	protein_coding
PRKAA2	protein_coding
PRKAA1	protein_coding
ELAVL1	protein_coding
BAP1	protein_coding
ABCC1	protein_coding
ACVR1B	protein_coding
TGFBR1	protein_coding
IFNG	protein_coding
ANO6	protein_coding
HMGB1	protein_coding
TNFAIP3	protein_coding
ATF3	protein_coding
ATM	protein_coding
YY1AP1	protein_coding
EGLN2	protein_coding
MIOX	protein_coding
TAZ	protein_coding
MTDH	protein_coding
IDH1	protein_coding
FBXW7	protein_coding
PANX1	protein_coding
DNAJB6	protein_coding
LONP1	protein_coding
SLC7A11	protein_coding
GPX4	protein_coding
AKR1C1	protein_coding
AKR1C2	protein_coding
AKR1C3	protein_coding
RB1	protein_coding
HSPB1	protein_coding
HSF1	protein_coding
NFE2L2	protein_coding

SQSTM1	protein_coding
NQO1	protein_coding
FTH1	protein_coding
MUC1	protein_coding
MT1G	protein_coding
SLC40A1	protein_coding
CISD1	protein_coding
HSPA5	protein_coding
ATF4	protein_coding
HELLS	protein_coding
SCD	protein_coding
FADS2	protein_coding
SRC	protein_coding
STAT3	protein_coding
PML	protein_coding
NFS1	protein_coding
TP63	protein_coding
CDKN1A	protein_coding
FH	protein_coding
CISD2	protein_coding
CBS	protein_coding
ISCU	protein_coding
ACSL3	protein_coding
OTUB1	protein_coding
CD44	protein_coding
BRD4	protein_coding
PRDX6	protein_coding
SESN2	protein_coding
NF2	protein_coding
ARNTL	protein_coding
HIF1A	protein_coding
JUN	protein_coding
CA9	protein_coding
TMBIM4	protein_coding
PLIN2	protein_coding
AIFM2	protein_coding
LAMP2	protein_coding
ZFP36	protein_coding
PROM2	protein_coding
CHMP5	protein_coding
CHMP6	protein_coding
CAV1	protein_coding
GCH1	protein_coding
PTGS2	protein_coding

DUSP1	protein_coding
NOS2	protein_coding
NCF2	protein_coding
MT3	protein_coding
UBC	protein_coding
ALB	protein_coding
TXNRD1	protein_coding
SRXN1	protein_coding
GPX2	protein_coding
BNIP3	protein_coding
OXSR1	protein_coding
SELENOS	protein_coding
ANGPTL7	protein_coding
DDIT4	protein_coding
ASNS	protein_coding
TSC22D3	protein_coding
DDIT3	protein_coding
JDP2	protein_coding
SLC1A4	protein_coding
PCK2	protein_coding
TXNIP	protein_coding
VLDLR	protein_coding
GPT2	protein_coding
PSAT1	protein_coding
LURAP1L	protein_coding
SLC7A5	protein_coding
HERPUD1	protein_coding
XBP1	protein_coding
SLC3A2	protein_coding
ZNF419	protein_coding
KLHL24	protein_coding
TRIB3	protein_coding
ZFP69B	protein_coding
ATP6V1G2	protein_coding
VEGFA	protein_coding
GDF15	protein_coding
TUBE1	protein_coding
ARRDC3	protein_coding
CEBPG	protein_coding
RGS4	protein_coding
BLOC1S5-TXNDC5	protein_coding
EIF2S1	protein_coding
HSD17B11	protein_coding
AGPAT3	protein_coding

SETD1B	protein_coding
FTL	protein_coding
TFRC	protein_coding
MAFG	protein_coding
DRD5	protein_coding
DRD4	protein_coding
MAP3K5	protein_coding
MAPK14	protein_coding
SLC2A1	protein_coding
SLC2A3	protein_coding
SLC2A6	protein_coding
SLC2A8	protein_coding
SLC2A12	protein_coding
SLC2A14	protein_coding
EIF2AK4	protein_coding
HBA1	protein_coding
NNMT	protein_coding
PLIN4	protein_coding
HIC1	protein_coding
STMN1	protein_coding
RRM2	protein_coding
CAPG	protein_coding
HNF4A	protein_coding
NGB	protein_coding
YWHAE	protein_coding
GABPB1	protein_coding
AURKA	protein_coding
RIPK1	protein_coding
PRDX1	protein_coding
NR1D2	protein_coding

Table S2. The clinical characteristics of colorectal cancer patients between the training and test sets.

Characteristics	Training set (*n*=354)		Test set (*n*=151)		Entire set (*n*=505)		*p*-value
	n	%	*n*	%	*n*	%	
Age(year)							0.767
<65	144	40.68%	64	42.38%	208	41.19%	
≥65	210	59.32%	87	57.62%	297	58.81%	
Sex							0.285
Female	155	43.79%	74	49.00%	228	45.35%	
Male	199	56.21%	77	51.00%	277	54.65%	
TNM stage							0.861
I	60	16.95%	30	19.87%	90	17.82%	
II	134	37.85%	54	35.76%	188	37.23%	
III	96	27.12%	44	29.14%	140	27.72%	
IV	54	15.26%	19	12.58%	73	14.46%	
Unknown	10	2.82%	4	2.65%	14	2.77%	
T stage							0.496
Tis	1	0.28%	0	0.00%	1	0.20%	
T1	12	3.39%	2	1.33%	14	2.77%	
T2	59	16.67%	32	21.19%	91	18.02%	
T3	244	68.93%	99	65.56%	343	67.92%	
T4	38	10.73%	18	11.92%	56	11.09%	
N stage							0.556
N0	206	58.19%	89	58.94%	295	58.41%	
N1	86	24.29%	37	24.50%	123	24.36%	
N2	62	17.52%	24	15.90%	86	17.03%	
Unknown	0	0.00%	1	0.66%	1	0.20%	
M stage							0.698
M0	261	73.73%	117	77.48%	378	74.85%	
M1	53	14.97%	19	12.58%	72	14.26%	
Unknown	40	11.30%	15	9.94%	55	10.89%	
Recurrence status							0.719
Non-recurrence	279	78.81%	122	80.79%	401	79.41%	
Recurrence	75	21.19%	29	19.21%	104	20.59%	
Survival status							0.146
Alive	277	78.25%	127	84.11%	404	80.00%	
Dead	77	21.75%	24	15.89%	101	20.00%	

Table S4. The clinical characteristics of patients between the high- and low-risk groups of the entire set.

Characteristics	High-risk group (*n*=184)		Low-risk group (*n*=321)		Entire set (*n*=505)		*p*-value
	n	%	*n*	%	*n*	%	
Age(year)							0.398
<65	71	38.59%	137	42.68%	208	41.19%	
≥65	113	61.41%	184	57.32%	297	58.81%	
Gender							0.405
Female	88	47.83%	141	43.93%	229	45.35%	
Male	96	52.17%	180	56.07%	276	54.65%	
TNM stage							0.026
I	24	13.04%	66	20.56%	90	17.82%	
II	62	33.70%	126	39.25%	188	37.23%	
III	57	30.98%	83	25.86%	140	27.72%	
IV	36	19.56%	37	11.53%	73	14.46%	
unknow	5	2.72%	9	2.80%	14	2.77%	
T Stage							0.001
Tis	1	0.54%	0	0.00%	1	0.20%	
T1	2	1.09%	12	3.74%	14	2.77%	
T2	23	12.50%	68	21.18%	91	18.02%	
T3	127	69.02%	216	67.29%	343	67.92%	
T4	31	16.85%	25	7.79%	56	11.09%	
N Stage							0.001
N0	90	48.91%	205	63.86%	295	58.41%	
N1	48	26.09%	75	23.37%	123	24.36%	
N2	46	25.00%	40	12.46%	86	17.03%	
unknow	0	0.00%	1	0.31%	1	0.20%	
M Stage							0.036
M0	128	69.56%	250	77.88%	378	74.85%	
M1	36	19.57%	36	11.22%	72	14.26%	
unknow	20	10.87%	35	10.90%	55	10.89%	
Recurrence status							0.004
Non-recurrence	133	72.28%	268	83.49%	401	79.41%	
Recurrence	51	27.72%	53	16.51%	104	20.59%	
Survival status							<0.001
Alive	116	63.04%	288	89.72%	404	80%	
Dead	68	36.96%	33	10.28%	101	20.00%	

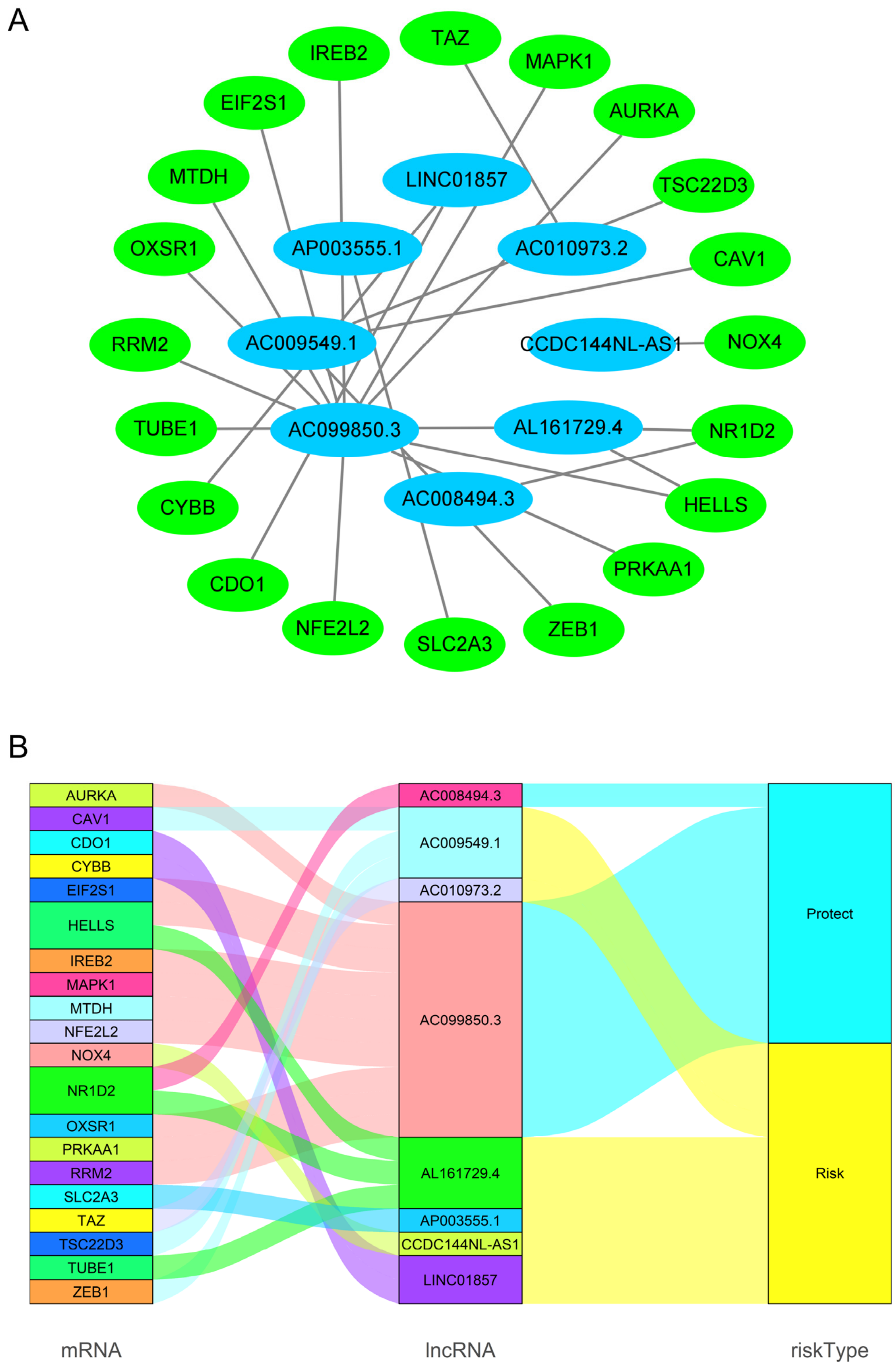

Figure S1. Co-expression network and Sankey relationship diagram between eight lncRNAs in the prognostic signature and their related mRNAs associated with ferroptosis. (A) A co-expression network comprised of 20 ferroptosis-related mRNAs and 8 lncRNAs. **(B)** The Sankey relationship diagram shows the relationship between 20 ferroptosis-related mRNAs and eight lncRNAs.

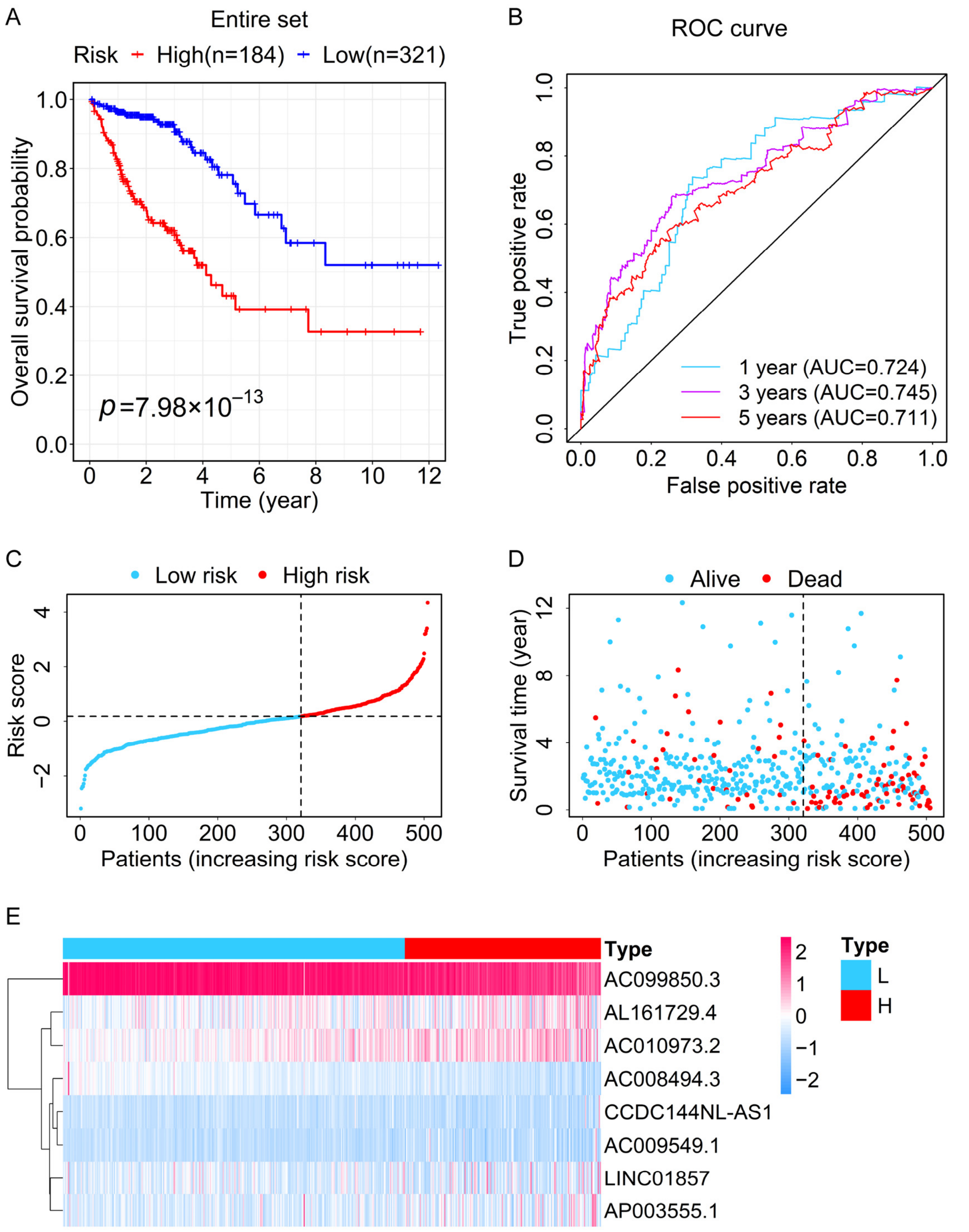

Figure S2. Verification for the prognostic value of the prognostic signature in the entire set. (A) Kaplan-Meier survival curve for OS of CRC patients in the high- and low-risk groups. **(B)** The time-dependent ROC curves of OS based on the risk score indicate the prognostic accuracy of the 8-lncRNAs signature. **(C)** The distribution of risk scores of CRC patients. **(D)** The scatter plot shows the overall survival time and survival status of CRC patients in the high-and low-risk groups. **(E)** Heatmap of clustering analysis for the expression of eight ferroptosis-related lncRNAs. L, low risk; H, high risk.

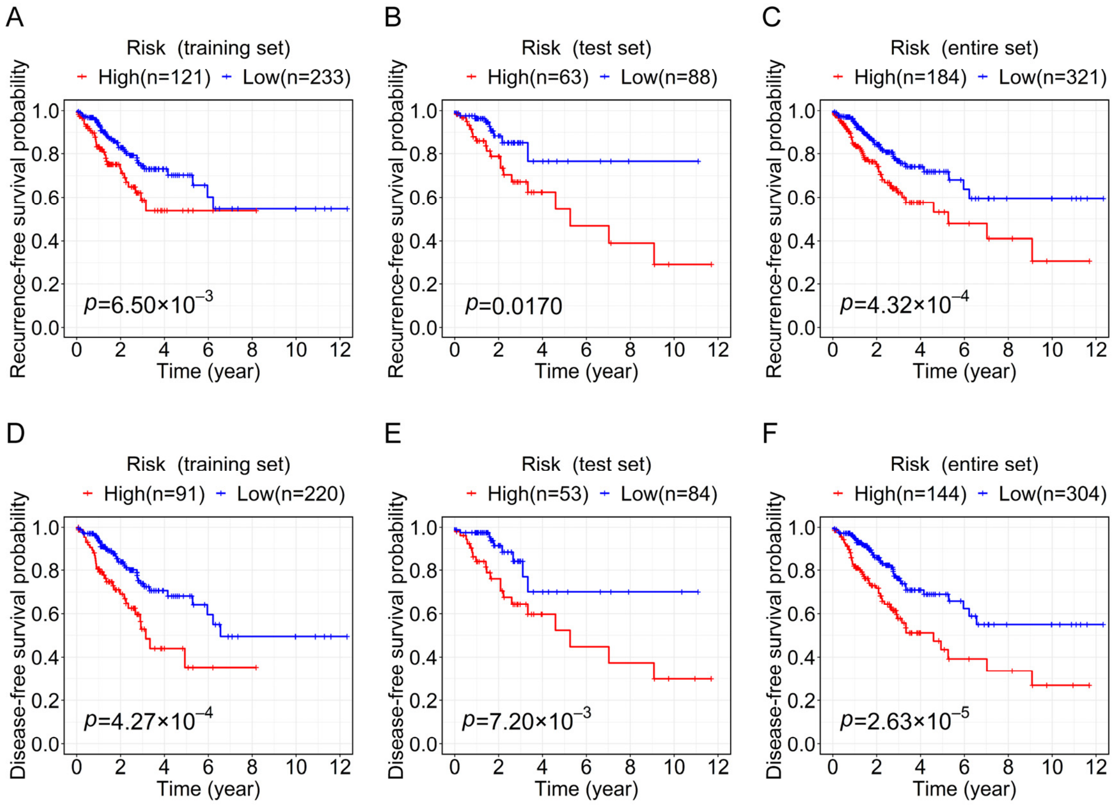

Figure S3. Evaluation and verification of the ferroptosis-related lncRNA prognostic signature for predicting RFS and DFS of CRC patients. The RFS curves for CRC patients in the high- and low-risk groups of the training set **(A)**, test set **(B)**, and entire set **(C)**. The DFS curves for CRC patients in the high- and low-risk groups of the training set **(D)**, test set **(E)**, and entire set **(F)**.

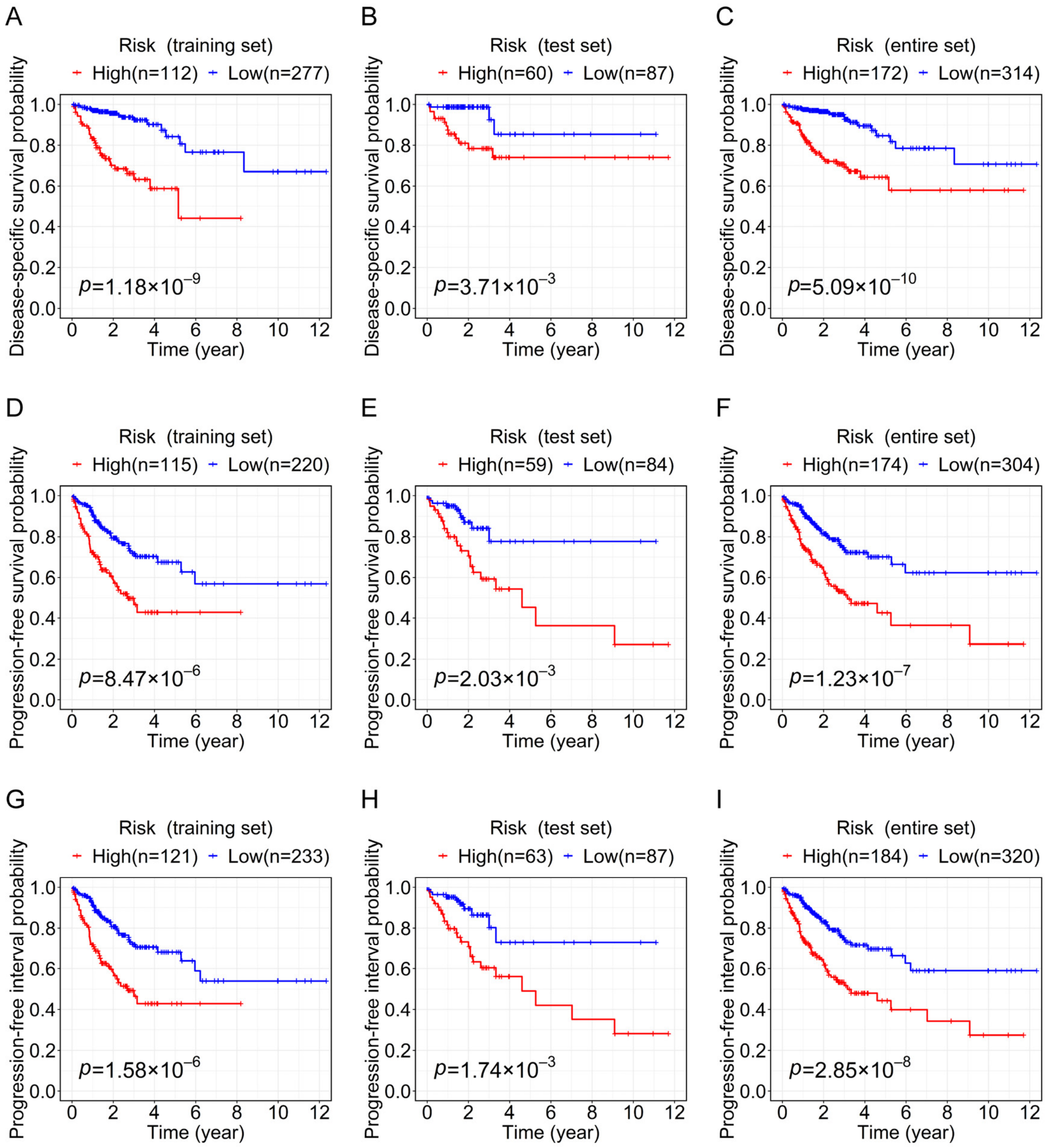

Figure S4. Evaluation and verification of the ferroptosis-related lncRNA prognostic signature for predicting DSS, PFS, and PFI of CRC patients. The DSS curves for CRC patients in the high- and low-risk groups of the training set **(A)**, test set **(B)**, and entire set **(C)**. The PFS curves for CRC patients in the high- and low-risk groups of the training set **(D)**, test set **(E)**, and entire set **(F)**. The PFI curves for CRC patients in the high- and low-risk groups of the training set **(G)**, test set **(H)**, and entire set **(I)**.

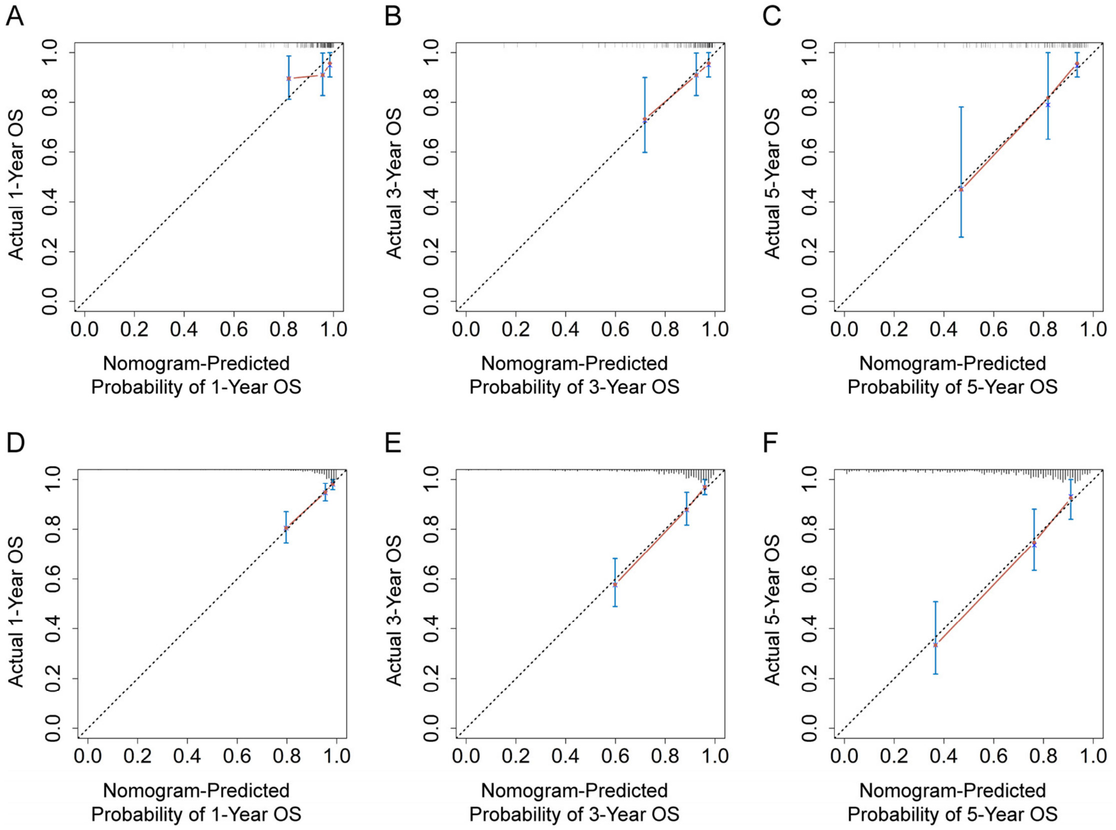

Figure S5. The calibration curves for the nomogram derived from the training set. The calibration curves at 1-year (**A**), 3-year (**B**), and 5-year (**C**) OS of the nomogram in the test set show the predicted result of the nomogram is close to the actual OS rate. The calibration curves at 1-year (**D**), 3-year (**E**), and 5-year (**F**) OS of the nomogram in the entire set show the predicted result of the nomogram is close to the actual OS rate.

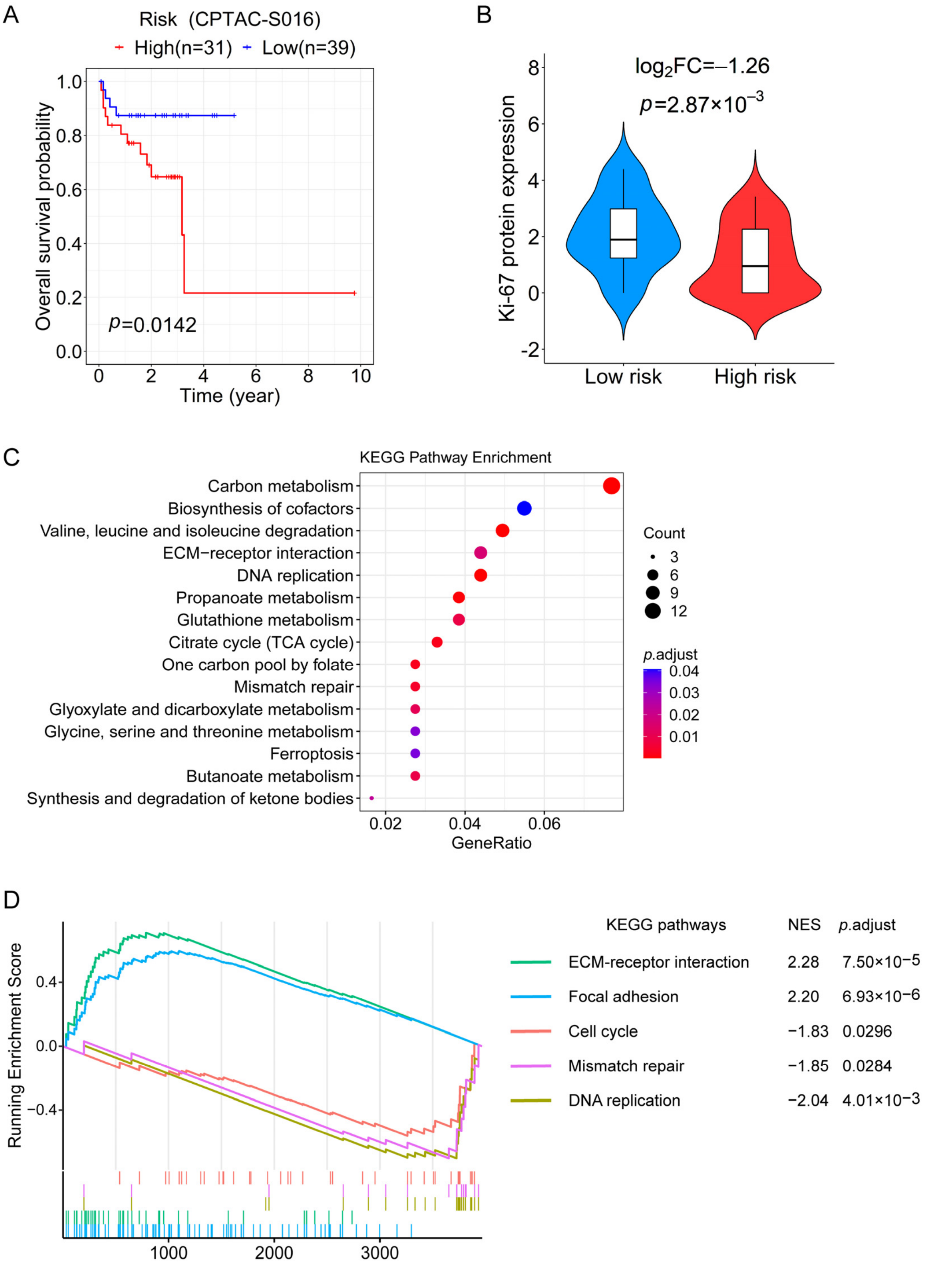

Figure S6. The functional enrichment analysis between the high- and low-risk groups in the proteomics dataset. (A) Kaplan-Meier survival curve analysis for OS shows the differences between the high- and low-risk groups in the proteomics dataset. **(B)** The violin plot shows the differential expression levels of Ki-67 protein analyzed by DESeq2 between the high- and low-risk groups in the proteomics dataset. **(C)** The significantly enriched KEGG pathways of the differentially expressed proteins between the high- and low-risk groups in the proteomics dataset. **(D)** The significantly enriched KEGG pathways between the high- and low-risk groups in the proteomics dataset through the GSEA analysis. log_2FC: log_2(fold change).
